# Ego-Lane Index Estimation Based on Lane-Level Map and LiDAR Road Boundary Detection

**DOI:** 10.3390/s21217118

**Published:** 2021-10-27

**Authors:** Baoguo Yu, Hongjuan Zhang, Wenzhuo Li, Chuang Qian, Bijun Li, Chaozhong Wu

**Affiliations:** 1 The 54th Research Institute of China Electronics Technology Group Corporation, Shijiazhuang 050081, China; yubg@sina.cn; 2 State Key Laboratory of Information Engineering in Surveying, Mapping and Remote Sensing, Wuhan University, Wuhan 430072, China; alvinlee@whu.edu.cn (W.L.); lee@whu.edu.cn (B.L.); 3 Engineering Research Center for Spatio-Temporal Data Smart Acquisition and Application, Ministry of Education of China, Wuhan University, Wuhan 430072, China; 4 Intelligent Transportation Systems Research Center, Wuhan University of Technology, Wuhan 430063, China; qian_c@whut.edu.cn (C.Q.); wucz@whut.edu.cn (C.W.)

**Keywords:** ego-lane index estimation, lane-level map, particle filter, road boundary detection, LiDAR, GNSS

## Abstract

Correct ego-lane index estimation is essential for lane change and decision making for intelligent vehicles, especially in global navigation satellite system (GNSS)-challenged environments. To achieve this, we propose an ego-lane index estimation approach in an urban scenario based on particle filter (PF). The particles are initialized and propagated by dead reckoning with inertial measurement unit (IMU) and odometry. A lane-level map is used to navigate the particles taking advantage of topologic and geometric information of lanes. GNSS single-point positioning (SPP) can provide position estimation with meter-level accuracy in urban environments, which can limit drift introduced by dead reckoning for updating the weight of each particle. Light detection and ranging (LiDAR) is a common sensor in an intelligent vehicle. A LiDAR-based road boundary detection method provides distance measurements from the vehicle to the left/right road boundaries, which provides a measurement for importance weighting. However, the high precision of the LiDAR measurements may put a tight constraint on the distribution of particles, which can lead to particle degeneration with sparse particle sets. To deal with this problem, we propose a novel step that shifts particles laterally based on LiDAR measurements instead of importance weighting in the traditional PF scheme. We tested our methods on an urban expressway at a low traffic volume period to ensure road boundaries can be detected by LiDAR measurements at most time steps. Experimental results prove that our improved PF scheme can correctly estimate ego-lane index at all time steps, while the traditional PF scheme produces wrong estimations at some time steps.

## 1. Introduction

Ego-lane index estimation refers to the determination of the lane index currently occupied by the vehicle, which is essential for intelligent vehicles, as it can recommend lane changes if needed. Visual multi-lane-marking detection is fully investigated in recent years. The driving lane and its adjacent lanes can be detected visually to infer which lane the vehicle locates. Zhao et al. [1] proposed a Catmull–Rom spline-based multi-lane detection and tracking system that could run in real time and detect both straight and curved shapes of lanes. Kang et al. [2] investigated multi-lane detection in highway scenarios based on geometric lane estimation by assuming that all lanes were parallel to each other and have the same width. Hur et al. [3] tried to dispose of this assumption and detect multiple lanes in urban driving environments using conditional random fields. They successfully detected non-parallel lanes found at intersections, in splitting lanes, and in merging lanes. However, since visual features vary with the illumination, weather condition and distance of the region, all above feature-based algorithm is restricted to the illuminant variation. To improve the robustness for extracting the multi-lane marking, some researchers proposed multi-lane detection algorithms based on neural networks. Chao et al. [4] detected multi-lanes based on a deep convolutional neural network. Huval et al. [5] used a convolutional neural network (CNN) architecture for highway scenarios. Neven et al. [6] built a network called LaneNet to distinguish each lane-border pixel. Garnett et al. [7] introduced a 3D LaneNet network architecture to predict the 3D layout of lanes from a single image. Although these neural-network-based methods performed well for multi-lane detection, they are limited in development by massive manually labeled datasets and extensive training on high-performance GPUs [8].

The digital map becomes more and more important for intelligent vehicles, which usually organizes index-aware lanes in tree data structures and supports spatial search of lanes. Map-enhanced sensor perception becomes a promising way for ego-lane index identification. A direct way to estimate the ego-lane index is localizing the vehicle precisely and then calculating the ego-lane index based on a lane-level map. To achieve accurate and consistent positioning performance, a common approach is to use the GNSS real-time kinematic (RTK) technique, but RTK often fails and results in serious errors owing to poor satellite geometry and disruption of radio signal reception because of skyscrapers in urban areas [9,10,11]. The map-matching method based on sensor data and a prior map is an alternative for precise localization. LiDAR sensor can achieve a high-precision position by matching LiDAR scans to a dense-point map [9,12,13,14]. However, the dense-point map requires expensive maintenance, as it is sensitive to seasonal changes and needs to be updated frequently. Some researchers used camera sensors to extract lane-related features and matched these features to a lane-level map to determine the vehicle pose and ego-lane index [15,16]. Lee et al. [17] integrated the ego-lane index information into the road-markings-based map-matching function using a DeepRoad network for ego-lane index identification. Choi et al. [18] identified in-lane localization using visual lane endpoints detection, the relation between a camera and a road, and the estimated vehicle’s orientation from a map. They identified ego-lane by generating a hypothesis about an ego-vehicle position per lane by the in-lane localization and verifying each hypothesis by matching the lane endpoints and an additional landmark such as a road sign with the map. These vision-and-map-based methods are more cost effective but need pixel-level benchmarks on camera images and time-consuming labeling processes to assess their performance [19].

A map-based probabilistic framework for ego-lane index estimation is another option. Rabe et al. [20] generated hypotheses of trajectory with map data and lane changes to describe several driving possibilities, aligned the hypotheses to the trajectory generated from vehicle odometry and yaw rate in a weighted least-squares sense, and finally determined the likelihood of the ego-vehicle being on a certain lane. They also proposed a PF framework to this ego-lane index estimation problem [21]. Visual lane detection was used for in-lane localization and radar objects projected onto a map to update the weights of particles. These two methods were compared by real driving conditions with a mix of one- to four-lane segments and found that the optimization-based approach performed better after intersections, whereas it often suffered from ambiguities on straighter stretches. The PF method was further extended for downtown scenarios with many lanes [22]. Specifically, a combination of weight update and sampling steps was proposed for the lane-marking distance observations to avoid diverging lane probability estimates. Ballardini et al. [23] also proposed a probabilistic framework for highway-like scenarios using a hidden Markov model (HMM) with a transient failure model, which considered inaccurate or missing road marking detections. They used GNSS measure and the number of lanes of the road, retrieved from a map service OpenStreetMap prior to enhancement of the visual ego-lane index estimation [24]. Similarly, Kasmi et al. [25] also used the lane information from OpenStreetMap to assist ego-lane determination and introduced a modular Bayesian network (BN) to infer the ego-lane from multiple inaccurate information sources. Svensson et al. [26] proposed a Bayesian filter to infer probability for the correct lane assignment by fusing lane-marking and vehicle detection data and map information.

All above works mainly relied on four sources of information: vision-based lane/road marking detection and radar-based adjacent vehicle detection, a map including lane geometry information, GNSS/IMU navigation data, and a filter method. Road boundary is an important map element and sensor-based road boundary detection can give us an estimate for the distances to the road boundaries, which is able to help infer ego-lane index. To take advantage of this information, in this paper, we proposed a PF framework using a LiDAR-based road boundary detection, a lane-level map, and GNSS/IMU/odometry navigation data. A lane-level map representation *Lanelets* was used in our work, where lanelets represent the drivable environment under both geometrical and topological aspects; more details about lanelets are presented in [27]. Navigation data and the distances to the road boundaries calculated from the LiDAR-based road boundary detection were used to update the particles. As the LiDAR-based road boundary detection can be determined accurately, it may put a tight constraint on the distribution of particles, which can result in sparse particle sets and performance deterioration. An important contribution of this paper is that we used the LiDAR measurement to shift particles laterally instead of updating the particle weight for each particle, which successfully avoids particle degradation.

## 2. Materials and Methods

**Figure 1:**
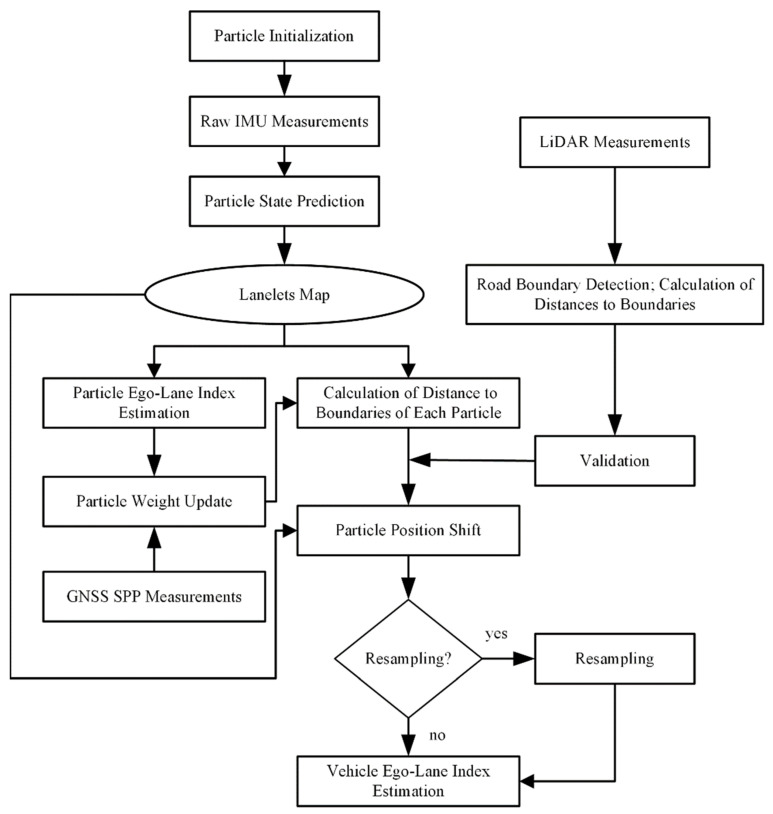
Flowchart of our method.

In this work, LiDAR, GNSS, IMU, wheel odometer measurements, and a lane-level map were fused by PF. Figure 1 shows the flowchart of our methods. Inertial sensor measurements were obtained at high sampling rates, which can produce high-frequency position and orientation information by the mechanization of the inertial navigation system (INS). Velocity measurement from odometry and INS mechanization were loosely coupled to achieve the real-time pose of the vehicle, which is called dead reckoning. GNSS SPP can provide real-time position estimation, but it only has meter-level positioning accuracy in urban areas because of signal blockages and reflections. Particles were initialized around the initial pose and predicted by dead reckoning at each time step. Based on the predicted position and orientation of each particle, taking advantage of road geometry and topology properties of the lane-level map, ego-lane index, and distance to left/right road boundaries could be calculated for each particle. Particle weight was updated by comparing its position with GNSS SPP measurement. The distance measurement from LiDAR-based road boundary detection was used to shift particle position laterally and estimate particle ego-lane index again. To avoid degenerate particle sets, a residual resampling was applied whenever the effective sample size became too low. Finally, the lanelet id with the highest sum of particle weights was chosen as the final ego-lane index.

Various coordinate systems were used in this work, including the following:

1. INS Navigation System (*n*-frame)

The *n*-frame is a local geodetic frame with platform mass center as the coordinate origin. The *X* axis pointing towards the east, the *Y* axis pointing to the north, and the *Z* axis completing a righthanded coordinate system. It is also called an east–north–up (ENU) system, forming a right-handed coordinate system.

2. World Coordinate System (*w*-frame)

The *w*-frame is used to express the GNSS positioning results. The initial GNSS position is taken as the origin. The *X*-*Y* plane is the local horizontal plane, with the *X* axis pointing east and the *Y* axis pointing north. The *Z* axis is perpendicular to the *X*-*Y* plane, forming a right-handed coordinate system. The *w*-frame is parallel to the *n*-frame.

3. LiDAR Coordinate System (*l*-frame)

The *l*-frame moves with the vehicle, with the LiDAR measurement center as the coordinate origin, the *X* axis pointing to the right along the LiDAR horizontal axis, the *Y* axis pointing forward along the LiDAR longitudinal axis, and the *Z* axis being perpendicular to the *X*-*Y* plane, forming a right-handed coordinate system.

4. Map Coordinate System (*m*-frame)

The discrete map points use the *w*-frame.

Transformations among these coordinate systems are assumed to be rigid-body transformations.

### 2.1. Digital Lane-Level Map

**Figure 2:**
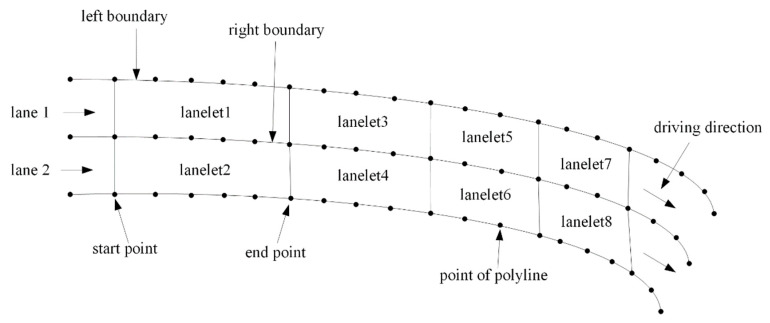
Structure of lanelets.

For lane-level navigation, we need a digital map containing geometric and topologic information of all available lanes to find the currently used lane. A lanelet map was used here, which was proposed by FZI Research Centre for Information Technology, Germany, in 2014 [27]. The lanelet map is organized by connected lanelets. A lanelet is defined by two polylines composed of a list of points as left and right boundaries, as shown in Figure 2. Except for the set of lanes and their interconnection, the lanelet map also contains the corresponding road traffic regulations, such as traffic signs, rules at intersections, and traffic lights. The vehicle must obey all the regulatory elements, which are linked to the lanelets when running on public roads. To make the spatial search in a lanelet map more efficient, R-tree is used to enable the spatial search for objects inside an arbitrary bounding box in *O*(log n).

In this work, we focused on measuring the distance of a pose to the bounds of a lanelet and getting the lanelet id of the pose, so that the regulatory elements were not considered. Neighboring and succeeding lanelets shared the same boundaries, and this information could determine their topologic relationship and the number of total parallel lanes in a certain road segment. To construct a lanelet map, we collected accurate positions in the *w*-frame of discrete points of polylines of all lanelets in our experimental area. The data collection vehicle mounted high-precision GNSS, IMU, LiDAR, and camera sensors; therefore, our map data had very high accuracy. Finally, the map data were structured by the liblanelet, as presented in [28].

### 2.2. Particle Initialization and Prediction

The vehicle starts with a known initial position $\left(b^{0},l^{0},h^{0}\right)$ in the *w*-frame, initial velocity $\left(v_{x}^{0},v_{y}^{0},v_{z}^{0}\right)$ in the *n*-frame, and initial attitude angles $\left(\gamma^{0},\theta^{0},\varphi^{0}\right)$. Particle state $X^{t}$ at the *t*th time step includes $\left(b^{t},l^{t},h^{t},v_{x}^{t},v_{y}^{t},v_{z}^{t},\gamma^{t},\theta^{t},\varphi^{t},ll^{t},d_{left}^{t},d_{right}^{t}\right)$, where $ll^{t}$ is the lanelet id where the vehicle locates in, which is calculated by function $f_{1}(b^{t},l^{t},h^{t},\varphi^{t})$ by searching for the *lanelet* map, $d_{left}^{t}$ and $d_{right}^{t}$ are the distances to the left and right road boundaries, which are calculated by function $f_{2}(b^{t},l^{t},h^{t},ll^{t})$ by fitting the polylines of boundaries as curves to get the distances from a point to curves. To initialize *N* particles, the *j*th particle $X_{j}^{0}$ is drawn from the initial probability density function (PDF) $p(X^{0})$, which is assumed to be a Gaussian distribution. Afterwards, at each time step $X_{j}^{t}$ is predicted by integrating INS and velocity from odometry.

INS mechanization could estimate the position, velocity, and attitude of a system from the raw data of the IMU if the initial pose is known. However, the estimation may contain large errors and drift rapidly because of the drift errors of the accelerometer and gyroscope. Performance of INS depends on IMU’s grade. Usually, a low-cost IMU has larger drift errors and the navigation solution deviates from ground truth in a short time period. More details about INS mechanization can be found in [29]. Real-time velocity measurement from odometry can compensate for the drift errors of INS to a certain extent. An error propagation model is used to fuse INS and velocity data to obtain a better navigation solution. The error propagation model via a first-order Taylor expansion is defined as follows:

(1) $$\begin{array}{l} \delta x_{j}=[\delta r_{j}^{n},\delta v_{j}^{n},\varepsilon_{j}^{n},\delta b_{j,a},\delta b_{j,g}]\\ \delta x_{j}^{t-}=\phi_{j}^{t-1}\delta x_{j}^{t-1}+u_{j}^{t-1} \end{array}$$

where the error state δx includes position error $\delta r^{n}$, velocity error $\delta v^{n}$, attitude error $\varepsilon^{n}$ in the *n*-frame, accelerometer drift $\delta b_{a}$, and gyroscope drift $\delta b_{g}$, $\phi_{t}$ is state transition matrix and $u_{t}$ is the driven response of the input white noise at time *t*. Velocity measurement is used to correct the error state δx via the extended Kalman filter (EKF) [30] and feed it back to the IMU mechanization for estimating the final navigation state. The EKF observation functions are

(2) $$\begin{array}{l} z_{j}^{t}=H\delta x_{j}^{t}+v_{j}^{t}\\ z_{j}^{t}=v_{j,IMU}^{n}-v_{odo}^{n} \end{array}$$

where *z* is the observation vector, *H* relates the error state vector and the measurements, *v* is a model error. Based on the prediction model in Equation (1), the error state vector corresponds to an error covariance *P*, which is predicted as

(3) $$P_{j}^{t-}=\phi_{j}^{t-1}P_{j}^{t-1}\phi_{j}^{t-1^{T}}+\Delta_{j}^{t-1}$$

EKF updates the error state vector as

(4) $$\begin{array}{l} \delta x_{j}^{t}=\delta x_{j}^{t-}+K(z_{j}^{t}-H\delta x_{j}^{t-})\\ P_{j}^{t}=(I-KH)P_{j}^{t-} \end{array}$$

where *K* is Kalman gain. Finally, $X_{j}^{t}$ can be estimated by

(5) $$\begin{array}{l} \left[b_{j}^{t},l_{j}^{t},h_{j}^{t}\right]=C_{n}^{w}\left[r_{j}^{n,t-}-\delta r_{j}^{n,t}\right]\\ \left[v_{j,x}^{t},v_{j,y}^{t},v_{j,z}^{t}\right]=C_{n}^{w}\left[v_{j}^{n,t-}-\delta v_{j}^{n,t}\right]\\ \left[\gamma_{j}^{t},\theta_{j}^{t},\varphi_{j}^{t}\right]=\left[\gamma_{j}^{t-},\theta_{j}^{t-},\varphi_{j}^{t-}\right]-\varepsilon_{j}^{n,t}\\ ll_{j}^{t}=f_{1}(b_{j}^{t},l_{j}^{t},h_{j}^{t},\varphi_{j}^{t})\\ \left[d_{j,left}^{t},d_{j,right}^{t}\right]=f_{2}(b_{j}^{t},l_{j}^{t},h_{j}^{t},ll_{j}^{t}) \end{array}$$

where $C_{n}^{w}$ is the rotation matrix.

In the scheme of PF, a weight is assigned to each particle $X_{j}^{t}$ to approximate the posterior PDF

(6) $$w_{j}^{t}=w_{j}^{t-1}\frac{p(z^{t}|X_{j}^{t})p(X_{j}^{t}|X_{j}^{t-1})}{q(X_{j}^{t}|X_{j}^{t-1},z^{t})}$$

where $p(z^{t}|X_{j}^{t})$ is the likelihood of observation $z^{t}$, $p(X_{j}^{t}|X_{j}^{t-1})$ is transition PDF, and $q(X_{j}^{t}|X_{j}^{t-1},z^{t})$ is proposal function. The calculation of weights of particles will be described in Section 2.4.

### 2.3. LiDAR-Based Road Boundary Detection

In this work, we used the LiDAR-based road boundary detection method proposed in [31]. This method provides a speed and accuracy tradeoff solution for road boundary detection in structured environments. Ground points are firstly segmented from raw LiDAR points by a RANSAC plane fitting method to separate off-ground and on-ground points since road boundaries are a part of the ground. Feature points are then extracted from the ground points. Three spatial features are used to extract the feature points: height difference between a point and neighbors, smoothness of the area around a point, and horizontal distance between two adjacent points. To classify the feature points as road boundary points, a road-segmentation-line-based method is used. After feature points are extracted and classified, there are still many false points, including vehicles, pedestrians, railway tracks, adjacent roads, etc. An iterative Gaussian process regression (GPR) algorithm is employed to model road boundary and filter out false points. Finally, the left and right boundaries are fitted as linear lines in the form of *ax* + *by* + *c* = 0 in the *l*-frame. To calculate the distance from the LiDAR to the road boundary, the closest point from the fitting line to the LiDAR is acquired.

This method is designed for point cloud from “Velodyne HDL-64E” LiDAR so that it contains some specific parameters that suit for “HDL-64E”, for example, the number of scan layers, the vertical azimuth of scanning layer, and the horizontal angular resolution. To apply this method to our “Velodyne HDL-32E” LiDAR, we changed the source codes to improve its availability. The source code can be found in [32].

As road boundary detection provides distance measurements for PF in our work, detection results should be validated. Three indicators were used: the coefficient *a* should be less than 0.005, the number of effective points after the iterative GPR algorithm should be larger than 20, and the difference between distances calculated at two adjacent time steps should be around v/f, where *v* is lateral velocity and *f* is frequency. If road boundary detection was invalid, we assumed that there were no distance measurements at the particle update step.

### 2.4. Particle Update and Ego-Lane Index Estimation

First, GNSS SPP with a meter-level positioning accuracy was used to update particle weight. The distance $d_{j}$ between the *j*th particle and the SPP position is calculated and compared with a threshold $d_{\max}$, which was determined by the positioning precision of SPP and set to 10 m in this work, resulting in a GNSS weight $w_{j}^{t}$

(7) $$w_{j,\mathrm{position}}^{t}=\left\{ \begin{array}{l} 0,\;if\;d_{j}>d_{\max}\\ w_{j}^{t-1},\;if\;d_{j}\leq d_{\max} \end{array}\right.$$

In a general PF scheme, the distances calculated from LiDAR road boundary detection can be used for the importance weighting with a Gaussian likelihood

(8) $$w_{j,\mathrm{LiDAR}}^{t}=\exp(-\frac{{\left(d_{left,\mathrm{LiDAR}}^{t}-d_{left,\mathrm{LiDAR}}^{t}\right)}^{2}+{\left(d_{right,\mathrm{LiDAR}}^{t}-d_{right,\mathrm{LiDAR}}^{t}\right)}^{2}}{2\delta_{\mathrm{distance}}^{2}})$$

where $\delta_{\mathrm{distance}}^{2}$ is the error variance of distance calculation from LiDAR road boundary detection, which is set to 0.1 in this work. Finally, for each particle, its overall weight $w_{j}$ is calculated as follows:

(9) $$w_{j}^{t}=w_{j,\mathrm{LiDAR}}^{t}w_{j,\mathrm{position}}^{t}$$

As the distance to the road boundaries can be determined very accurately by LiDAR, the likelihood in Equation (8) can be tight, compared to the particle distribution after the prediction step. High weights will be assigned to only a few particles, and many particles will be deleted in the next resampling step. Generally, this is the desired effect, as the filter may converge quickly. However, the likelihood in Equation (8) is tight only in the lateral position but arbitrarily wide in the longitudinal position. In this case, the few surviving particles may not represent the actual distribution. Inspired by [22], we used a different approach in this work.

Distance measurements were used to update the particle position instead of particle weights. Since the distances from the vehicle to road boundaries were known, we shifted each particle laterally such that its lateral position matched the distance measurements. New distances became

(10) $$\begin{array}{l} d_{j,left}^{t’}=d_{left,\mathrm{LiDAR}}^{t}+\xi_{left},\;\xi_{left}\sim Ν(0,\delta_{\mathrm{distance}}^{2})\\ d_{j,right}^{t’}=d_{right,\mathrm{LiDAR}}^{t}+\xi_{right},\;\xi_{right}\sim Ν(0,\delta_{\mathrm{distance}}^{2})\\ \left(b_{j}^{t’},l_{j}^{t’},h_{j}^{t’})=f_{3}(d_{j,left}^{t’},d_{j,right}^{t’}\right) \end{array}$$

where function $f_{3}$ can calculate position from distance measurements via geometrical calculation using the map. New lanelet id is calculated by new position $\left(b_{j}^{t’},l_{j}^{t’},h_{j}^{t’}\right)$ with function $f_{1}(b^{t},l^{t},h^{t},\varphi^{t})$. As lane widths are stipulated by regulations, and they can be known in advance, if the lane number of a road is known, the road width is fixed. Therefore, either $d_{j,left}^{t’}$ or $d_{j,right}^{t’}$ is used in Equation (10) in practice, and the other one is calculated by road width. A residual resampling, which eliminates particles with low weights while copies particles with high weights, is applied whenever the effective sample size becomes too low. Finally, the lanelet id with the highest sum of particle weights is chosen as the final lane index.

## 3. Experimental Results

**Figure 3:**
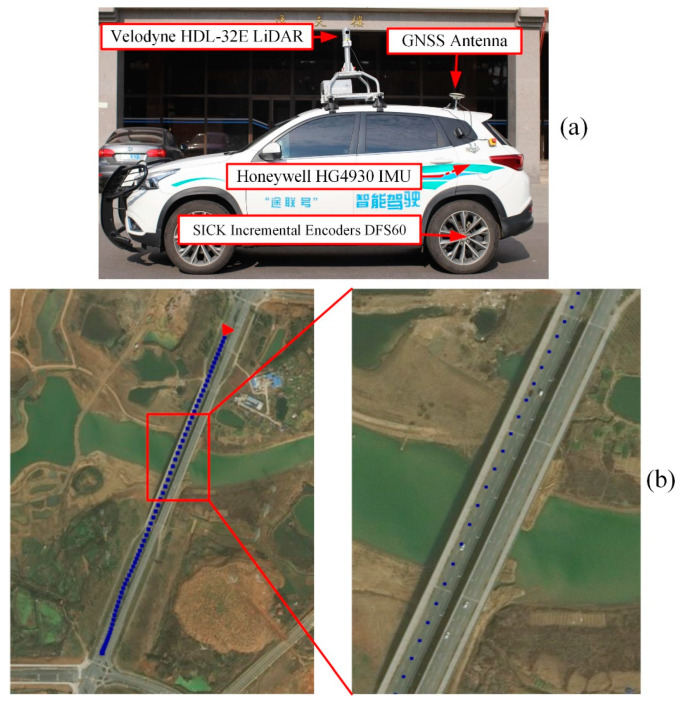
The vehicle and sensors used in our work (**a**) and an overhead satellite image of the test region (**b**). GNSS RTK trajectory of the test route is denoted by blue dots. The red arrow shows the starting point and the driving direction. To be more specific, the area in the red frame is zoomed in.

Field experiments were carried out to test our PF framework of ego-lane index estimation. The test vehicle we used is a Chery Tiggo car equipped with GNSS operating at 1 Hz, IMU at 600 Hz, and LiDAR at 10 Hz (shown in Figure 3a). “Trimble BD982” was used for GNSS positioning, which supports BDS/GPS/Galileo/GLONASS and multiple GNSS frequencies. “Velodyne HDL-32E” LiDAR used in this work has a horizontal angle range [0°, 360°] and a vertical angle range [−30°, 10°] with 32 scans. “Honeywell HG4930” is a very high-performance micro-electromechanical system (MEMS) IMU, and its angular and velocity random walk are $0.04^{\circ }/\sqrt{h}$ and $0.3\;\mu g/\sqrt{Hz}$, respectively. The odometry was “SICK Incremental encoders DFS60”, which was mounted on the left rear wheel of the vehicle. The encoders can measure 2048 pulse per revolution so that they can produce high-precision velocity measurements.

Our experimental field was on the Huashan road in Wuhan, China with an open-sky environment for GNSS. Therefore, both GNSS RTK and SPP can achieve high-precision positioning estimation. In order to evaluate the performance of our method in dense urban environments, where GNSS positioning is poor because buildings block, reflect, and diffract the signals, we simulated a high-occlusion environment (satellite signals with elevation angle in [0, 40°] are blocked, and random noises are added to pseudo-range measurements). GNSS RTK results from the open-sky environment are regarded as ground truth positions of vehicles with centimeter-level accuracy, which is denoted with blue dots in Figure 3b.

**Figure 4:**
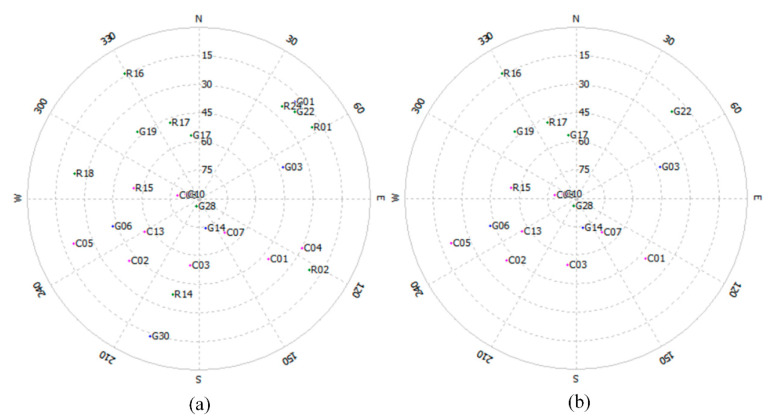
Sky plot of visible satellites of GPS + BDS in the open-sky environment (**a**) and in the simulated high-occlusion environment (**b**) at the first epoch.

**Figure 5:**
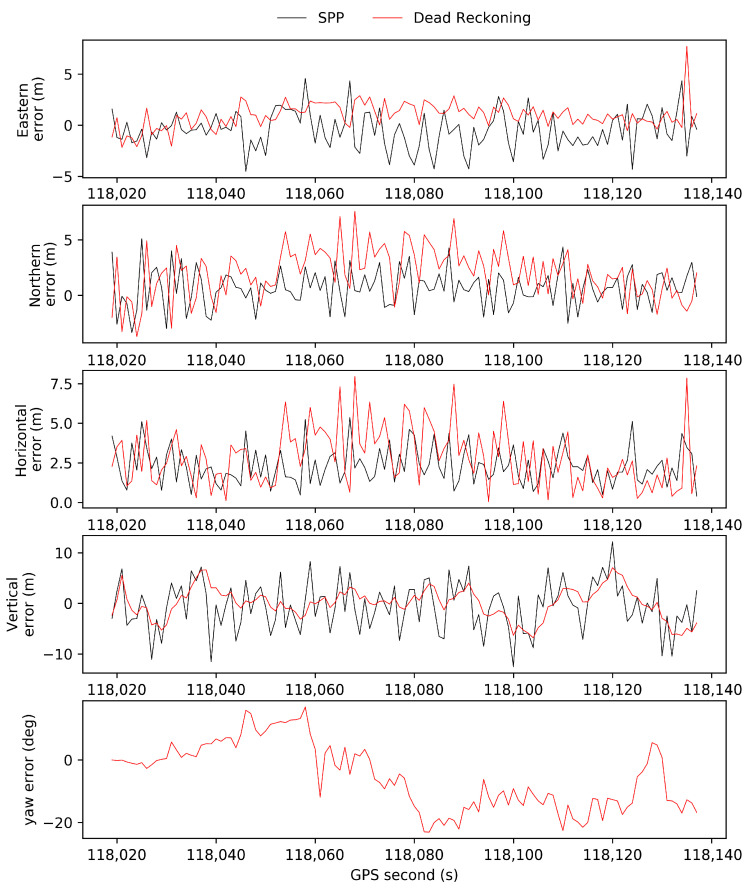
Positioning errors in the eastern, northern, horizontal, and vertical directions of GNSS SPP in the simulated high-occlusion environment and the INS/odometry dead reckoning.

SPP results from the simulated high-occlusion environment provide real-time GNSS measurements for our method. Figure 4 shows the sky plot of visible satellites of GPS + BDS + GLONASS above the platform’s horizon at the first epoch. In the simulated high-occlusion environment, the satellites with elevation angles lower than 40° are eliminated, and the positioning precision becomes a few meters. Figure 5 shows the positioning errors and yaw errors in the *n*-frame of GNSS SPP results, and dead reckoning results in the high-occlusion environment by comparison with the ground truth of GNSS RTK in the open-sky environment. We can observe that horizontal errors of SPP can be as large as 5 m and exhibit lateral positioning deviations of up to a few lanes. The vertical accuracy is about 2 times worse than the horizontal accuracy because the GNSS receiver can only track satellites in two quadrants of its vertical plane which does not provide the same strength of geometric calculations [33]. The positioning precision of dead reckoning is a little worse than the GNSS SPP measurement. Yaw angle is essential for the calculation of lanelet id as shown in Equation (5). Therefore, yaw angle errors from dead reckoning are also shown in the figure and the errors increased within time to up to −20°.

**Figure 6:**
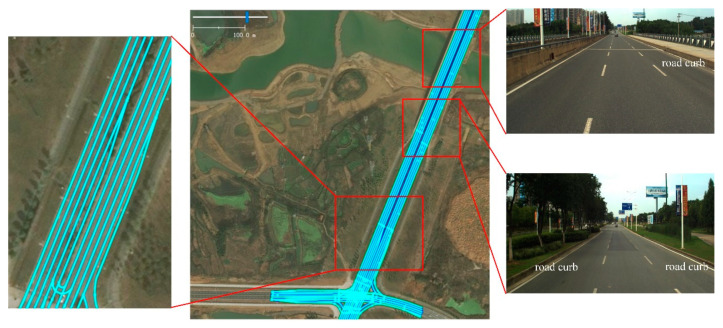
A part of the digital lane-level map used in our work; the area in the red frame is zoomed in.

**Figure 7:**
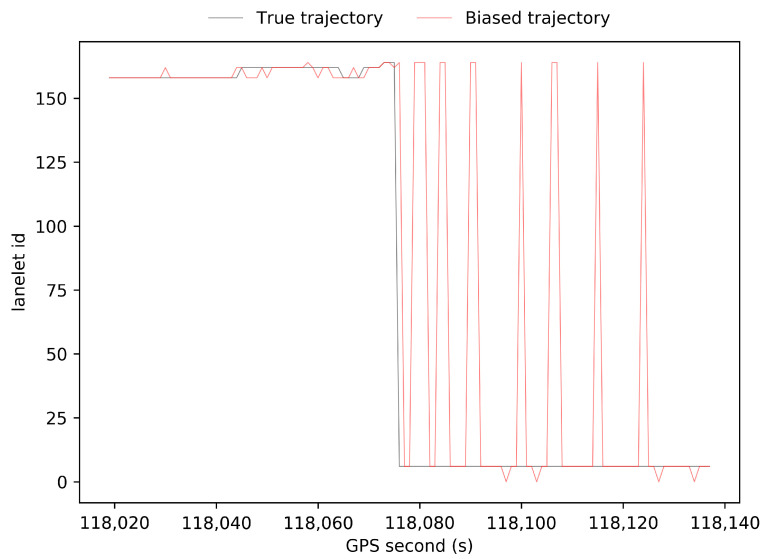
Evolution of lanelet ids of the true trajectory and the biased trajectory from dead reckoning.

Figure 6 shows a part of our digital lane-level map, and the lines in the map represent the polylines of the left and right boundaries of lanes. Accurate discrete points in the polylines are acquired by high-precision navigation and IMU equipment. The points are then organized with the lanelet library. There are about 20 lanelets in our test region. The test road includes three or four parallel lanes, as shown in the right images in Figure 6. Our trajectory changes lanes frequently in purpose. Figure 7 shows the lanelet ids at each time step calculated by the true trajectory of GNSS RTK in the open-sky environment and by the biased trajectory from the dead reckoning. It can be seen that ego-lane index estimation is wrong at many time steps because of biased positions and yaw angles, and the vehicle is assumed to be off-road at some time steps, with lanelet id being 0, which is dangerous for intelligent vehicles, as it may result in wrong decisions.

**Figure 8:**
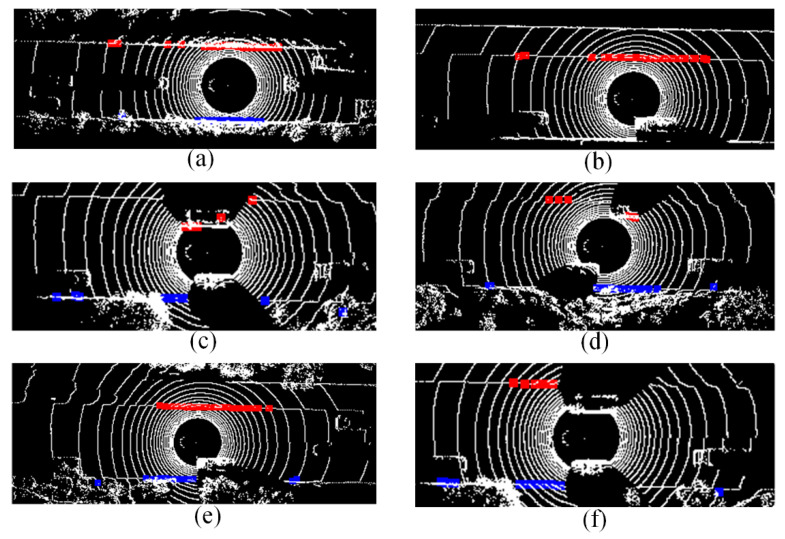
Some results of LiDAR-based road boundary detection and (**a**–**f**) show different scenarios; red dots mean left road boundary, and blue dots represent right road boundary.

From the right images in Figure 6, we can also infer that the road is highly structured with road curbs. There is a bridge in the trajectory which contains a road curb on one side. In other places of the trajectory, the curbs are distributed on both sides. Therefore, the LiDAR-based road boundary detection method performs well in our work. In most places of the trajectory, both left and right road boundaries are detected correctly, as shown in Figure 8a. However, the moving vehicles on the road disturbed the detection results as road curbs cannot be correctly extracted from the LiDAR points as shown in Figure 8b–f. In Figure 8b, only one boundary is detected, which can be used in Equation (10) as the LiDAR measurement for the left boundary. In Figure 8c,d, the left and right road boundaries are detected, but they are not used for Equation (10), as they do not satisfy the three indicators described in Section 2.3—the coefficients of the fitting lines of left road boundaries are larger than 0.005, and the number of effective points for the right road boundaries is less than 20. For some cases, road boundaries are still effectively detected despite moving obstacles, as shown in Figure 8e,f, in which either left or right road boundary detection satisfies the indicators. To avoid the bad effects of the moving vehicles on the LiDAR-based road boundary detection, we chose a low traffic-volume period.

**Figure 9:**
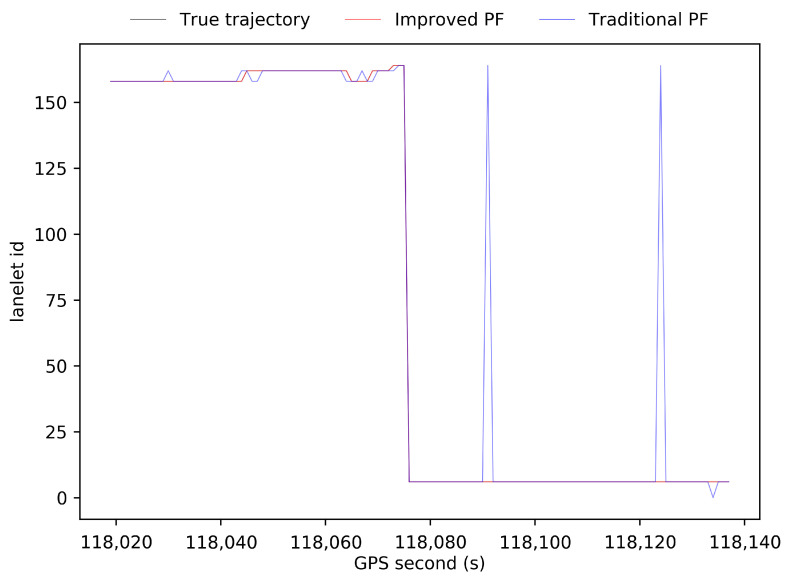
Evolution of lanelet ids of the true trajectory, the results from our improved PF scheme, and the results from the traditional PF scheme.

**Figure 10:**
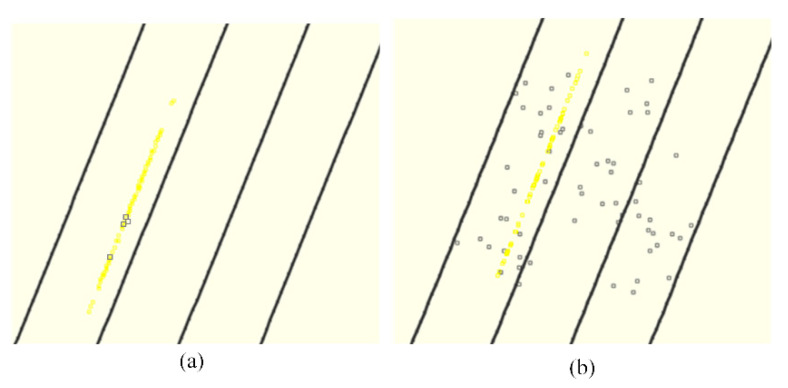
Distribution of particles on the lane-level map: (**a**) shows the particles at the 50th time step after update in our improved PF scheme (yellow squares) and in the traditional PF scheme (black squares); (**b**) shows the particles at the 3rd time step before update (black squares) and after update (yellow squares) in our improved PF scheme.

Figure 9 shows the lanelet ids at each time step calculated by the ground truth of GNSS RTK in the open-sky environment, by the results of our improved PF scheme, and by the results of the traditional PF scheme in the simulated high-occlusion environment. Compared to the results of the biased trajectory from dead reckoning in Figure 7, both PF schemes have positive effects on the ego-lane index estimation, taking advantage of distance measurements from LiDAR detection. Our improved PF scheme finds the correct lane index at all time steps, but there are still some wrong estimations in the results of the traditional PF scheme. Figure 10a shows the distribution of particles at the 50th time step after update by Equation (10) in our improved PF scheme (yellow squares) and the particles at the 50th time step after update by Equation (8) in the traditional PF scheme (black squares). First, 100 particles are randomly initialized around the true location over a four-lane road segment following a uniform distribution over the whole segment. Then, lateral distances to road boundaries are observed and importance weight updating according to the likelihood shown in Equation (8) is applied repeatedly. The particle set converges to the black squares shown in Figure 10a after 50 steps of prediction, importance weight updating, and resampling. One can see that there are only four particle sets are left and particle degradation occurs. The diversity of particles is worse and badly represents the posterior PDF of the position of the vehicle based on sensor measurements, which leads to the wrong estimations of ego-lane.

Our improved PF scheme relieves this problem by shifting each particle laterally such that its lateral position meets the distance calculation from LiDAR detection instead of importance weight updating. Using this approach, the particle distribution evolves to the one shown with yellow squares in Figure 10a after 50 filter steps. Figure 10b shows particles after prediction with black squares and after lateral shift with yellow squares at the third time step. We can deduce that the longitudinal distribution of particles represents the posterior probability much better than with the traditional importance weighting: the 100 particles (yellow squares in Figure 10a) spread rather uniformly along the road, while before the update (black squares in Figure 10a), they converge to only a few particle sets, leaving large areas without any particles. This meets the knowledge from LiDAR road boundary detection which puts strong constraint in the lateral direction but no constraint in the longitudinal direction. Therefore, our approach keeps the diversity of particles and leads to a more stable and reproducible behavior of the PF without increasing the number of particles.

## 4. Conclusions and Discussion

In this work, a novel PF scheme for ego-lane index estimation was presented with sensors that are readily available in an intelligent vehicle: GNSS, IMU, odometry, and LiDAR sensors, combined with a lane-level map which is organized with a lanelet library. The lane index is presented as lanelet id. In total, 100 particles were initialized and propagated via dead reckoning by IMU and odometry. In urban scenarios, GNSS signals may be blocked by high buildings or trees leading to meter-level positioning accuracy, which is insufficient for the intelligent vehicle. However, it can limit the drift of dead reckoning. A LiDAR-based road boundary detection proposed in [31] was applied for calculating distances from the vehicle to left and right road boundaries. The high-precision distance measurement puts a tight constraint on particles in the lateral direction while putting arbitrarily a wide constraint in the longitudinal direction. The traditional importance weight updating process in PF can hardly deal with this problem, leading to particle degradation. A novel update step was proposed by shifting particles laterally to place corresponding to the distance measurements so that particle distribution can correctly represent the posterior PDF of the position of the vehicle. Together with the support of topologic and geometric information of lanes from the map, a reliable ego-lane index estimation could be performed. Field experiments prove that our method can correctly estimate the ego-lane index during the trip.

Our system greatly relies on accurate road boundary detection which gives important inference of the lateral position where a vehicle locates on the map. However, the road boundary detection method used in this paper is badly affected by moving obstacles, as LiDAR points hit the obstacles rather than road curbs so that positions of curbs become unknown. In future works, we will focus on solving road boundary detection under traffic jams, for example, using deep learning to predict the position of the road curb when it is blocked by moving obstacles. To make our approach more practicable, we will try low-cost MEMS and test our approach with more scenarios, such as downtown scenarios with complex intersections.

## Data Availability

Not applicable.
